# Sugar transport from sheaths to seeds: A role for the kinase SnRK1

**DOI:** 10.1093/plphys/kiac187

**Published:** 2022-05-02

**Authors:** Marieke Dubois

**Affiliations:** Department of Plant Biotechnology and Bioinformatics, Ghent University, Ghent, Belgium; VIB Center for Plant Systems Biology, Ghent, Belgium

Rice (*Oryza sativa*) is an important cereal crop that provides up to 60% of the calories taken up by half of the population worldwide (Slewinski, 2012). To feed the growing population, it is crucial for biologists and plant breeders to understand the mechanisms that control rice yield, which is not only determined by the number of grains per panicle, but also by the biomass of each grain. Grain biomass increases during reproductive development by importing sugars into the seeds, a process called grain filling.

Plants accumulate sugars in photosynthetic organs during vegetative growth and store them as nonstructural carbohydrates (NSC), of which starch is the best-known example. Long-term NSC storage occurs in the stem of most grass species, including rice, maize (*Zea mays*), sugarcane (*Saccharum Officinarum*), and sorghum (*Sorghum bicolor*; Slewinski, 2012). In some rice varieties, NSC are also stored in the sheath, the lower region of the leaf enrobing the stem (Okamura et al., 2018). NSC are converted to soluble sugars and transported to the energy-demanding developing seeds during grain filling (Figure 1). Despite the importance of NSC remobilization during grain filling in rice, the molecular regulators that control sheath-to-panicle sugar transport are still largely unknown.

**Figure 1:**
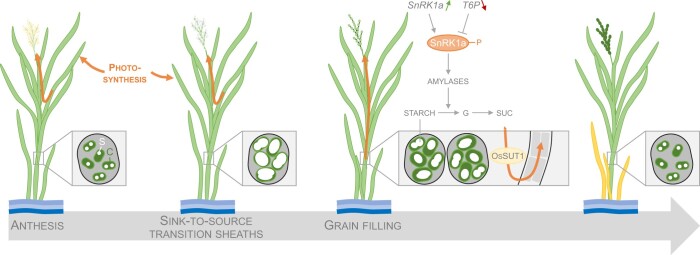
Schematic representation of the role of SnRK1 in carbohydrate remobilization in Nipponbare rice sheaths. During vegetative growth and up to 10 days following anthesis, leaves and sheaths accumulate carbohydrates via photosynthesis. Excess carbon is stored as large starch granules in rice sheaths. During grain filling, nonstructural carbohydrates from the sheaths are remobilized to the panicles in a SnRK1-dependent manner. SnRK1a gene expression is induced, SnRK1a proteins accumulate, and their catalytic domain is phosphorylated for activation. Levels of the SnRK1-inhibiting compound T6P decrease. Finally, the main sucrose transporter in sheaths, *Oryza sativa SUCROSE TRANSPORTER1 (OsSUT1)*, is transcriptionally activated. S, starch; C, chloroplast; G, glucose; SUC, sucrose. Arrows on the plants represent the carbon flow.

In the current issue of *Plant Physiology*, Hu et al. (2022) present a study on NSC remobilization during grain filling in Nipponbare, a rice cultivar with a sequenced genome. Using a detailed time-course analysis to measure multiple physiological and biochemical parameters during the period following anthesis, Hu et al. found that the sheath is a major source of NSC for grain filling. During vegetative development, sheaths accumulated large amounts of sugars, stored as massive starch granules in the chloroplasts (Figure 1). Peak starch accumulation in sheaths and sheath biomass was detected approximately 10 days after anthesis, after which the sheaths become a carbon source tissue and redistribute NSC to sugar-demanding organs. Interestingly, in Nipponbare, the decrease of biomass and starch content in sheaths was more pronounced than in stems, suggesting an important role for sheaths in sugar remobilization during grain filling.

To study NSC sheath-to-grain transport, the authors developed an assay that artificially induces NSC remobilization. Cutting the plant’s leaves triggered a rapid activation of starch-degrading amylases and the *Oryza sativa* *SUCROSE TRANSPORTER1*, which is one of the main transporters in sheaths and actively pumps sucrose into the phloem (Chen and Wang, 2008; Scofield et al., 2009). Consequently, starch content in sheaths decreased rapidly upon leaf cutting, validating that the leaf-cutting assay can induce the physiological processes mimicking those occurring during grain filling.

In Arabidopsis (*Arabidopsis thaliana*), one of the most important kinases regulating sugar availability and distribution between organs/tissues is Sucrose nonfermenting1-RELATED PROTEIN KINASE1 (SnRK1). SnRK1 fine-tunes sucrose availability by activating a transcription factor that induces expression of amylases and by regulating key enzymes in sucrose synthesis. In rice, two subfamilies of SnRK1, SnRK1a, and SnRK1b, have been identified. Hu et al. found that the expression and protein abundance of *SnRK1a* increases in sheaths when sheaths transition from sugar sinks to sugar sources (Figure 1). Similar changes in *SnRK1a* were also observed with the leaf-cutting assay. Subsequently, the authors investigated possible factors regulating SnRK1 activity during grain filling. On the one hand, the SnRK1 catalytic subunit requires phosphorylation to be active, and leaf cutting increased SnRK1 phosphorylation. On the other hand, high levels of trehalose-6-phosphate (T6P) can reduce SnRK1 activity. During grain filling or following leaf cutting, levels of T6P decreased in the sheaths. Altogether, the biochemical data support a model in which SnRK1 abundance and activity are increased during grain filling in rice (Figure 1).

To validate that SnRK1 participates in NSC remobilization from sheaths during grain filling, the authors generated a *snrk1a* mutant. By using the CRISPR–Cas9 (Clustered Regularly Interspaced Short Palindromic Repeats–CRISPR-associated protein 9) system, they generated a truncated SnRK1a protein lacking the C-terminal regulatory domain of an important SnRK1 subunit. In this mutant, SnRK1 activity in the sheaths decreased, sheaths contained more starch, and plants displayed incomplete grain filling. Seed setting rate, an important parameter determining rice yield, was also drastically reduced. That the grain filling process is clearly affected in the *snrk1a* mutants underscores the importance of SnRK1 in sheath-to-panicle NSC remobilization during grain filling in rice.

Finally, the authors used the CRISPR-edited *snrk1a* mutants to identify SnRK1 targets via phosphoproteomics at different time points during grain filling. About 600 unique phosphosites were present to a different extent in the wild type versus the *snrk1a* mutant. Some of the enriched motifs corresponded to the well-known SnRK1 target sites. The authors clustered the identified phosphosites based on their occurrence over time and further analyzed the clusters that showed a clear decrease or increase in abundance during grain filling. Using this elegant approach, they found clusters enriched for proteins related to carbohydrate catabolism and NSC transport.

Taken together, the phenotypical and biochemical analyses presented in Hu et al. (2022) offer insights in the process of grain filling in rice. SnRK1, an essential kinase for plant development and growth, was shown to play a critical role in NSC remobilization from sheaths to grain. The identification of such a central regulator opens possibilities for biotechnological engineering of rice with increased yields (Slewinski, 2012). For example, engineering faster NSC remobilization from the sheaths post floral initiation could redirect energy use from shoot growth toward grain filling, improving grain filling of all grains of the panicle (Mullet, 2017). This method has been successful in other cereals, such as maize, where altering T6P metabolism reduced shoot growth but increased seed yield (Nuccio et al., 2015). The possibility to initiate, and potentially also complete grain filling in a reduced time lapse, is of particular importance for rice plants growing in suboptimal climates where drought stress often threatens the yield at the end of the season.

## Funding

M.D. is a postdoctoral fellow of Flanders Research Foundation (FWO-12Q7919N).

*Conflict of interest statement*. None declared.
